# Tumor-infiltrating B cells affect the progression of oropharyngeal squamous cell carcinoma via cell-to-cell interactions with CD8^+^ T cells

**DOI:** 10.1186/s40425-019-0726-6

**Published:** 2019-10-17

**Authors:** Kamila Hladíková, Vladimír Koucký, Jan Bouček, Jan Laco, Marek Grega, Miroslav Hodek, Michal Zábrodský, Milan Vošmik, Kateřina Rozkošová, Hana Vošmiková, Petr Čelakovský, Viktor Chrobok, Aleš Ryška, Radek Špíšek, Anna Fialová

**Affiliations:** 1 SOTIO a.s, Jankovcova 1518/2, CZ-17000 Prague 7, Prague, Czech Republic; 2 Department of Immunology, 2nd Faculty of Medicine, Charles University and University Hospital Motol, Prague, Czech Republic; 3 Department of Otorhinolaryngology and Head and Neck Surgery, 1st Faculty of Medicine, Charles University and University Hospital Motol, Prague, Czech Republic; 4 The Fingerland Department of Pathology, Charles University Faculty of Medicine in Hradec Králové and University Hospital Hradec Králové, Hradec Králové, Czech Republic; 5 Department of Pathology and Molecular Medicine, 2nd Faculty of Medicine, Charles University and University Hospital Motol, Prague, Czech Republic; 6 Department of Oncology and Radiotherapy, Charles University Faculty of Medicine in Hradec Králové and University Hospital Hradec Králové, Hradec Králové, Czech Republic; 7 Department of Otorhinolaryngology and Head and Neck Surgery, Charles University Faculty of Medicine in Hradec Králové and University Hospital Hradec Králové, Hradec Králové, Czech Republic

**Keywords:** HNSCC, Tumor-infiltrating B lymphocytes, HPV

## Abstract

**Background:**

Standard treatment of oropharyngeal squamous cell carcinoma (OPSCC) is associated with high morbidity, whereas immunotherapeutic approaches using PD-1:PD-L1 checkpoint blockade only show moderate response rates in OPSCC patients. Therefore, a better stratification of patients and the development of novel therapeutic protocols are crucially needed. The importance of tumor-infiltrating B cells (TIL-Bs) in shaping antitumor immunity remains unclear; therefore, we analyzed frequency, phenotype, prognostic value and possible roles of TIL-Bs in OPSCC.

**Methods:**

We utilized transcriptomic analysis of immune response-related genes in 18 OPSCC samples with respect to human papillomavirus (HPV) status. The density and localization of CD20^+^, CD8^+^ and DC-LAMP^+^ cells were subsequently analyzed in 72 tissue sections of primary OPSCC samples in relation to patients’ prognosis. The immunohistochemical approach was supplemented by flow cytometry-based analysis of phenotype and functionality of TIL-Bs in freshly resected primary OPSCC tissues.

**Results:**

We observed significantly higher expression of B cell-related genes and higher densities of CD20^+^ B cells in HPV-associated OPSCC samples. Interestingly, CD20^+^ TIL-Bs and CD8^+^ T cells formed non-organized aggregates with interacting cells within the tumor tissue. The densities of both intraepithelial CD20^+^ B cells and B cell/CD8^+^ T cell interactions showed prognostic significance, which surpassed HPV positivity and CD8^+^ TIL density in stratification of OPSCC patients. High density of TIL-Bs was associated with an activated B cell phenotype, high CXCL9 production and high levels of tumor-infiltrating CD8^+^ T cells. Importantly, the abundance of direct B cell/CD8^+^ T cell interactions positively correlated with the frequency of HPV16-specific CD8^+^ T cells, whereas the absence of B cells in tumor-derived cell cultures markedly reduced CD8^+^ T cell survival.

**Conclusions:**

Our results indicate that high abundance of TIL-Bs and high density of direct B cell/CD8^+^ T cell interactions can predict patients with excellent prognosis, who would benefit from less invasive treatment. We propose that in extensively infiltrated tumors, TIL-Bs might recruit CD8^+^ T cells via CXCL9 and due to a highly activated phenotype contribute by secondary costimulation to the maintenance of CD8^+^ T cells in the tumor microenvironment.

**Electronic supplementary material:**

The online version of this article (10.1186/s40425-019-0726-6) contains supplementary material, which is available to authorized users.

## Background

Oropharyngeal squamous cell carcinoma (OPSCC) forms a specific subset of head and neck squamous cell carcinoma (HNSCC), associated in up to 90% of patients with human papillomavirus (HPV) infection [1, 2]. A positive HPV-status has been reported to correlate with better locoregional control, a longer overall survival [3, 4] and a higher immunogenicity of the tumor [5, 6]. The immune response has been suggested as a key factor in the better outcome of patients with HPV-associated tumors [7].

Indeed, in a wide range of malignancies, characterization of the adaptive immune response has been shown to be a valid prognostic tool for improving the stratification of the patients compared to the current staging system [8–12]. During the past two decades, extensive immuno-oncology research has been mainly focused on T cells and several studies have reported the association between a high density of tumor infiltrating T lymphocytes (TILs) and increased patient survival [8, 11–13]. Consequently, most of the recent immunotherapeutic approaches target T cell-mediated immunity. In 2016, the immune checkpoint inhibitors pembrolizumab and nivolumab were approved by the American Food and Drug Administration (FDA) for HNSCC patients whose disease has progressed during or after platinum-based chemotherapy. However, clinical trials with the above mentioned PD-1:PD-L1 targeting agents only reported modest response rates (13–23%) in HNSCC patients [14–17]. Therefore, novel immunotherapy targets and consequent effective therapeutic strategies are still needed for this type of carcinoma.

In contrast to T cells, the role of B cells in the tumor microenvironment remains controversial. Both positive and negative impacts of B cells on tumor immunity and disease progression have been reported [18, 19]. Most of the studies concerning mouse models assign B cells a tumor-promoting character, whereas studies of human solid tumors mainly associated a high density of tumor-infiltrating B cells (TIL-Bs) with a favorable clinical outcome [20–24]. It has been proposed that TIL-Bs generate antitumor antibodies [20, 25, 26], produce antitumor cytokines, exert direct cytotoxicity towards tumor cells and are capable to present tumor-associated antigens (TAA) [19, 27–30].

It has been hypothesized that TAA-specific T cells are primed in tumor-draining lymph nodes and subsequently migrate to the tumor tissue [31, 32]. However, in addition to the primary DC-T cell interactions in the lymph nodes, secondary interactions with activated APCs at the target tissue site are needed for the generation of an effective immune response. Indeed, especially in cases of viral infections, T cell interactions with antigen-experienced activated DCs and/or B cells at the site of infection have been shown to be essential for the secondary recall and long-term survival of T cells [33–35]. Therefore, TIL-Bs might act as local APCs essential for the secondary stimulation of tumor-specific T cells.

In this study, we assessed the frequency, distribution and phenotype of TIL-Bs in OPSCC samples. For the first time, we showed significant differences between patients with low versus high infiltrates of CD20^+^ B cells not only in the clinical outcome but also in the activation status of TIL-Bs and the density of tumor-infiltrating HPV 16 E6/E7-specific CD8^+^ T cells. Our results indicate that in immunologically “hot” OPSCCs, highly activated TIL-Bs may provide crucial secondary costimulatory stimuli to the tumor-infiltrating CD8^+^ T cells, resulting in the maintenance of CD8^+^ T cell-mediated antitumor immunity and prolonged patient survival.

## Materials and methods

### Patients and samples

#### Cohort 1

Formalin-fixed paraffin-embedded (FFPE) primary OPSCC specimens were obtained from 72 patients who underwent radical surgery at the University Hospital Hradec Kralove in Czech Republic between 2001 and 2014. All of the patients underwent surgical resection of the primary tumor using external approach with therapeutic neck dissection, followed by postoperative radiotherapy. Concomitant chemotherapy was applied in 30.5% (*n* = 22) of patients.

#### Cohort 2

Primary fresh OPSCC tissues and matching FFPE tumor sections were obtained from 21 patients after therapeutic surgery at the University Hospital Motol in Prague, Czech Republic, between August 2015 and May 2016.

#### Cohort 3

Fresh primary OPSCC specimens and blood samples were obtained from 21 patients immediately after therapeutic surgery at the University Hospital Motol in Prague, Czech Republic, between March 2018 and June 2019. Control tonsils were obtained from 6 healthy donors.

None of the patients enrolled in this study had received any neoadjuvant chemo- or radiotherapy. The pathological staging of OPSCC was reviewed and classified by an experienced pathologist according to the 8th edition of the American Join Committee on Cancer. The clinical-pathological characteristics of the patients are summarized in Table 1.

**Table 1:** Clinico-pathological characteristics of the patients

Variable	Cohort No. 1		Cohort No. 2		Cohort No. 3	
	No.	%	No.	%	No.	%
Total No. of Patients	72		21		21	
Age						
Median	57		63		63	
Range	41–76		40–73		41–75	
Sex						
Male	55	76.4	16	76.2	13	61.9
Female	17	23.6	5	23.8	8	38.1
Tumor site						
Palatine tonsil	62	86.1	18	85.7	14	66.7
Base of tongue	10	13.9	2	9.5	4	19.0
Oropharynx NS	0	0	1	4.8	3	14.3
T status						
T1	17	23.6	6	28.6	6	28.6
T2	36	50.0	11	52.4	12	57.1
T3	13	18.1	4	19	3	14.3
T4	6	8.3	0	0	0	0
N status						
N0	1	1.4	4	19	1	4.8
N1	15	20.8	17	81	16	76.2
N2	53	73.6	0	0	4	19.0
N3	3	4.2	0	0	0	0
Stage 8th edition of AJCC						
I	46	63.9	17	81	15	71.4
II	13	10.1	4	19	6	28.6
III	5	6.9	0	0	0	0
IV	8	19.1	0	0	0	0
Stage 7th edition of AJCC						
I	0	0	1	4.8	1	4.8
II	0	0	3	14.3	2	9.6
III	18	25	4	19.0	1	4.8
IV	54	75	13	61.9	17	80.8
HPV status						
HPV+	63	87.5	21	100	21	100
HPV-	9	12.5	0	0	0	0
Smoking history						
Smoker	22	30.6	7	34.4	9	42.9
Ex-smoker	25	34.7	5	23.8	2	9.5
Non-smoker	25	34.7	9	42.8	10	47.6

### TaqMan low-density array

Total RNA was isolated from 1 × 10^6^ tumor-tissue derived cells using the RNA Easy Mini Kit (Qiagen) according to the manufacturer’s instructions. The concentration and purity of the samples were determined by spectrophotometry with a NanoDrop© 2000c (Thermo Scientific), and the RNA integrity was assessed using a 2100 Bioanalyzer (Agilent). Complementary DNA was synthesized from 100 ng of total RNA using the High Capacity RNA-to-cDNA Kit (Applied Biosystems). The gene expression of immune response-associated genes was determined using TaqMan low-density array (TLDA) cards according to the manufacturer’s instructions (Applied Biosystems). The TLDA cards (TaqMan® Array Human Immune Panel) were run on a Viia7 instrument (Applied Biosystems) using TaqMan® Universal Master Mix II, no UNG (Applied Biosystems). Ct values were analyzed using GenEx software (MultiD Analyses). Relative gene expression levels were calculated using the ΔΔCt method and were normalized to the expression levels of reference genes GUSB and TFRC, selected by GeNorm from 6 reference genes assessed in total.

### Immunohistochemistry

Staining was carried out on FFPE sections following deparaffinization and antigen retrieval. Endogenous peroxidase was blocked with 3% hydrogen peroxide. The sections were incubated with protein block (DAKO) and stained with primary antibodies against CD8 (SP16, Spring Bioscience), CD20 (L26, Dako) and DC-LAMP (1010E1.01, Dendritics), followed by the manifestation of enzymatic activity and hematoxylin counterstaining. The images were acquired using a Leica Aperio AT2 scanner (Leica).

### Quantification of tumor-infiltrating immune cells

**Fig. 1:**
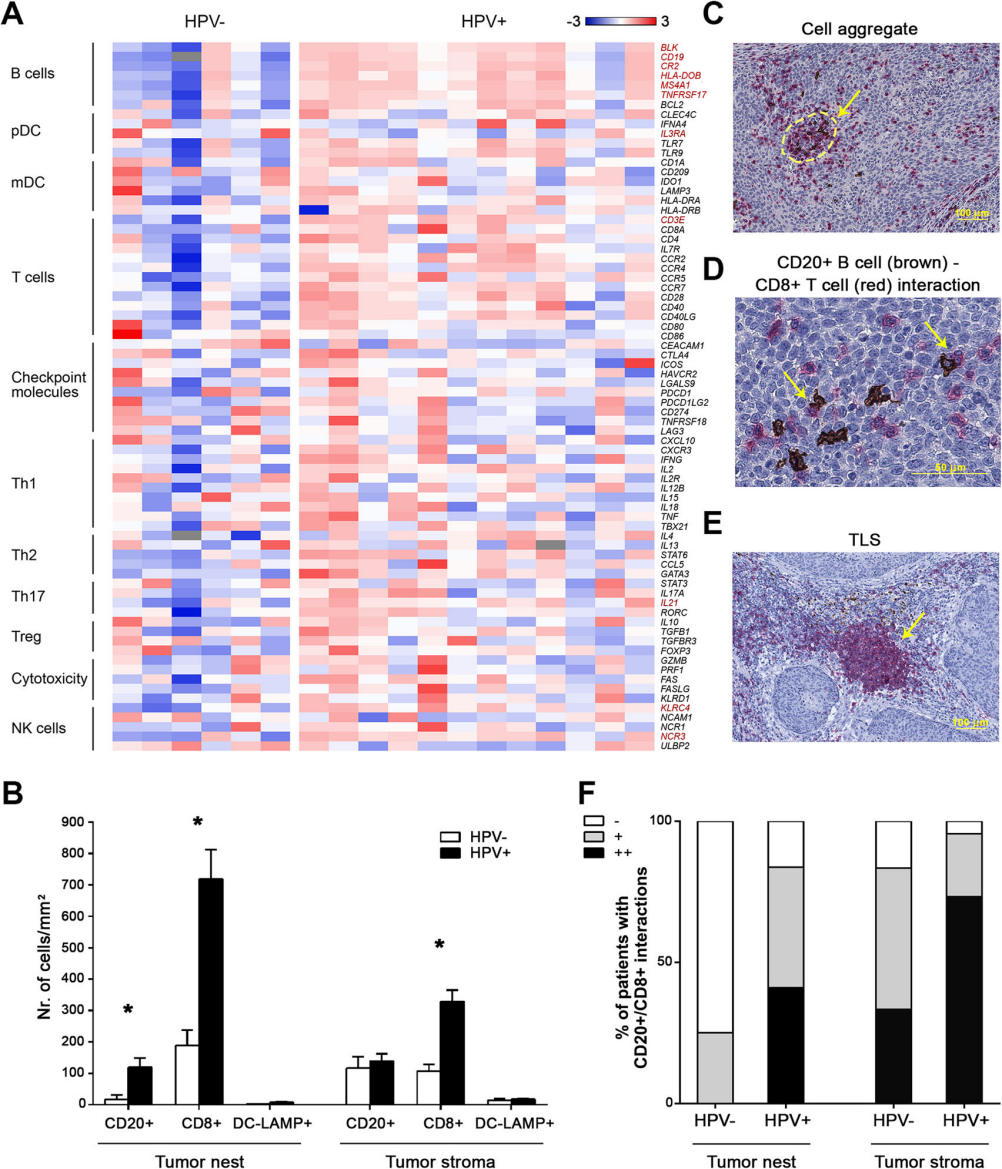
Differences in frequencies of tumor-infiltrating leucocytes in patients with oropharyngeal squamous cell carcinoma (OPSCC) with respect to the HPV status. **a** The heat-map expresses z-scores of relative mRNA expression of indicated genes within HPV- (*n* = 6) and HPV+ (*n* = 12) tumor samples. Genes with significantly different expression in HPV- and HPV+ tumors are marked in red. **b** Columns represent the mean (+ standard error of mean, SEM) densities of CD20^+^ B cells, CD8^+^ T cells and DC-LAMP^+^ dendritic cells in tumor nests and tumor stroma of immunohistochemically stained FFPE sections of OPC patients from Cohort 1 (*n* = 72). **c** Non-organized CD20^+^ B cell (brown)/CD8^+^ T cell (red) aggregate. **d** CD20^+^ B cell (brown) – CD8+ T cell interactions. **e** TLS with germinal center. **f** Columns show proportions of patients with detected B cell/CD8^+^ T cell interactions in the tumor nest and the tumor stroma of OPC tissue sections (−, interactions not detected; +, interactions detected in 1–5 visual fields; ++, interactions in > 5 visual fields). * *p* < 0.05 (Mann-Whitney U test)

Each section was scanned and evaluated for immune cell infiltration in the tumor nest and tumor stroma in 10 representative visual fields at 10× magnification using a Ventana Image Viewer. The cell numbers were related to tumor nest/tumor stroma area assessed by Calopix software (Tribvn). Additionally, a semiquantitative analysis of CD20^+^/CD8^+^ cell-cell interactions was performed (−, negative sections; +, sections positive for B cell/CD8^+^ T cell interactions in 1–5 visual fields; ++, sections positive for interactions in > 5 visual fields). The cell-cell interaction was defined as a direct cell-cell contact of CD20^+^ B cell and CD8^+^ T cell (Fig. 1d) within an aggregate of 20–100 cells (Fig. 1c) or in a distance up to 100 μm from a margin of the aggregate. The quantification was performed by two independent observers and reviewed by an experienced pathologist.

### Processing of fresh tumor tissues and blood samples

Fresh tumor tissues were mechanically and enzymatically digested as described previously [6]. Subsequently, the specimens were passed through a 100-μm nylon cell strainer (BD Biosciences) and washed with PBS. Peripheral blood mononuclear cells (PBMCs) were isolated from the peripheral blood samples by centrifugation on a Ficoll-Paque density gradient (GE Healthcare).

### Flow cytometry

Single cell suspensions derived from tumor tissues were labeled using a panel of monoclonal antibodies as listed in Additional file 1: Table S1. For the intracellular detection of cytokines and Ki-67, the cells were fixed and permeabilized with the Fixation/Permeabilization Buffer Set (eBioscience) and intracellularly labeled with primary antibodies. The cells were analyzed on a BD LSR Fortessa (BD Biosciences) and evaluated with FlowJo software (TreeStar).

### Detection of HPV-specific T cells

The detection of HPV16 E6/E7-specific T cells was performed as described previously [36]. Briefly, freshly prepared tumor-derived single cell suspensions were seeded at a concentration of 3 × 10^5^ cells/ml into 24 well plate and TILs were expanded for two weeks in the presence of IL-2. Monocytes from autologous PBMCs were isolated using the Human CD14 Positive Selection Kit (Stemcell Technologies), loaded with HPV16 E6 and E7 peptide pools (5 μg/ml) (JPT) and added to expanded TILs at a ratio of 1:10. After 6 h of incubation with Brefeldin A (BioLegend), the cells were stained with antibodies for the intracellular detection of IFNγ.

### Analysis of T cell viability and functional capacity

Tumor-derived single cell suspensions were split into halves. One half was depleted of B cells using CD19 MicroBeads (Miltenyi Biotech) according to the manufacturer’s instructions. The second half was subjected to the same procedures without addition of CD19 MicroBeads. After magnetic separation, cell suspensions (6 × 10^5^ cells/ml) were cultured in RPMI 1640 supplemented with 10% heat-inactivated FCS, L-glutamine and penicillin-streptomycin (Invitrogen) in 48 well plates for 6 days without any additional stimuli. The viability of CD4^+^ and CD8^+^ T cells and their capacity to produce cytokines was assessed at day 1 and 6 using LIVE/DEAD™ Fixable Blue Dead Cell Stain Kit (Invitrogen) and intracellular cytokine staining as described above.

### Detection of cytokines and chemokines in cell culture supernatants

Tumor-derived single cell suspensions (1 × 10^6^ cells/ml) were cultured in RPMI 1640 supplemented with 10% heat-inactivated FCS, L-glutamine and penicillin-streptomycin (Invitrogen). For some of the patient samples (*n* = 3), the B cells were depleted from the cell suspensions using CD19 MicroBeads (Miltenyi Biotech) according to the manufacturer’s instructions. To detect the concentrations of lymphotoxin, IFNγ, TNFα, IL-6, IL-10, IL-12, CXCL9 and CXCL13 released into the culture supernatant, the MILLIPLEX™ Human Cytokine Kit (Merck) was used according to the manufacturer’s instructions.

### HPV detection

#### Immunohistochemical analysis

The antibody against p16INK4a (Purified Mouse Anti-Human p16, Clone G175–405, BD Pharmingen TM, dilution 1:100) or the CINtec Histology Kit (Roche) was used. The intensity of staining and the proportion of stained cells were evaluated. Samples positive for p16 expression showed more than 70% of positive cells and revealed nuclear and/or cytoplasmic staining.

#### PCR

HPV DNA from the paraffin-embedded tissue was extracted with the MagCore Genomic DNA FFPE One-Step Kit (RBC Bioscience) according to the manufacturer’s protocol.

HPV DNA detection and genotyping were performed by qualitative real-time PCR with the AmoyDx Human Papillomavirus Genotyping Detection Kit (Amoy Diagnostics). The test is designed for the specific amplification of the L1 gene in HPV DNA to detect and genotype 19 high-risk HPVs and 2 low-risk HPVs (HPV 6 and 11). The sensitivity of the test is 100 copies of HPV DNA per reaction. An internal control is provided in the assay to test for sample quality and the presence of inhibiting factors.

HPV DNA^+^/p16^+^ samples were considered HPV-positive.

### RNA extraction from isolated CD8^+^ T cells and quantitative real time PCR

CD8^+^ T cells were isolated from tumor tissue-derived single cell suspensions and PBMC using the EasySep™ Human CD8 Positive Selection Kit II (StemCell Technologies). Total RNA was isolated from 1 × 10^6^ CD8^+^ T cells using the RNA Easy Mini Kit (Qiagen) according to the manufacturer’s instructions. The concentration and purity of the samples were determined by spectrophotometry with a NanoDrop© 2000c (Thermo Scientific), and the RNA integrity was assessed using a 2100 Bioanalyzer (Agilent). Complementary DNA was synthesized from 100 ng of total RNA using the iScript cDNA Synthesis Kit (BIO-RAD). The gene expression levels of BCL2L1, IL-2, IL-2R, CD27, CD40L, and the β-actin housekeeping gene were evaluated using the CFX 96™ Real-Time System (BIO-RAD). The specificity of the amplified PCR product was assessed using an Agilent DNA 1000 Kit (Agilent). The relative expression of the target genes was normalized to the expression of β-actin.

### Statistical analysis

Statistical analyses were performed using Statistica® 10.0 software (StatSoft). The differences between HPV-positive and HPV-negative tumor samples were analyzed using the Mann-Whitney U test. The prognostic value of tumor-infiltrating immune cells was analyzed using the log-rank test. Additionally, the Cox proportional hazard model was used to perform univariate and multivariate analyses of possible prognostic factors. Only variables with significant differences observed in the univariate analysis were included in the multivariate analysis. The correlation between the presence of B cell/CD8+ T cell interactions and HPV positivity/presence of HPV16 E6/E7-specific CD8^+^ T cells was evaluated using Pearson’s chi-square test. Variability in proportions of Ki-67^+^ cells was detected using Kruskal-Wallis ANOVA. Differences in the B cell phenotype were analyzed using one-way ANOVA, followed by Tukey’s post hoc test. The results were considered statistically significant when *p* < 0.05.

## Results

### HPV-associated tumors show significantly higher densities of CD20^+^ B cells and CD8^+^ T cells in comparison to HPV-negative samples

To evaluate the transcriptional signature of immune response-related genes in HPV-associated and HPV-negative tumors, we assessed the expression of selected genes using TaqMan analysis. Tumor samples with a positive HPV status expressed significantly higher levels of all of the B cell-related genes analyzed, namely, *BLK, CD19, CR2, HLA-DOB, MS4A1* and *TNFRSF17* (Fig. 1a).

To supplement the results of gene expression, we immunohistochemically analyzed the density of CD20^+^, CD8^+^ and DC-LAMP^+^ cells in 72 OPSCC tumor tissue sections (Cohort 1). Compared to HPV-negative tumors, HPV-associated tumors showed significantly higher infiltrates of CD20^+^ B cells in the tumor nest and significantly higher levels of CD8^+^ T cells in both the tumor nest and the tumor stroma. No differences were observed in DC-LAMP expression (Fig. 1b). Additionally, we observed that tumor-infiltrating CD20^+^ B cells and CD8^+^ T cells create non-organized aggregates in both the tumor nests and the tumor stroma (Fig. 1c) with CD20^+^ B cells and CD8^+^ T cells in a direct cell-cell interaction (Fig. 1d). The proportion of these cell-cell interactions was markedly higher in HPV-associated tumors than in HPV-negative tumors (Fig. 1f). In contrast to direct CD20^+^ B cell/CD8^+^ T cell interactions, no differences between HPV-associated and HPV-negative samples were observed in the density of tertiary lymphoid structures (TLS) with germinal centers (Fig. 1e). Well-defined TLS with germinal centers were detected in 29.8% of HPV-associated samples and in 25.0% of HPV-negative samples.

### High densities of CD20^+^ B cells, CD8^+^ T cells and CD20^+^ B cell/CD8^+^ T cell interactions in the tumor nest are positive prognostic factors in OPSCC patients

**Fig. 2:**
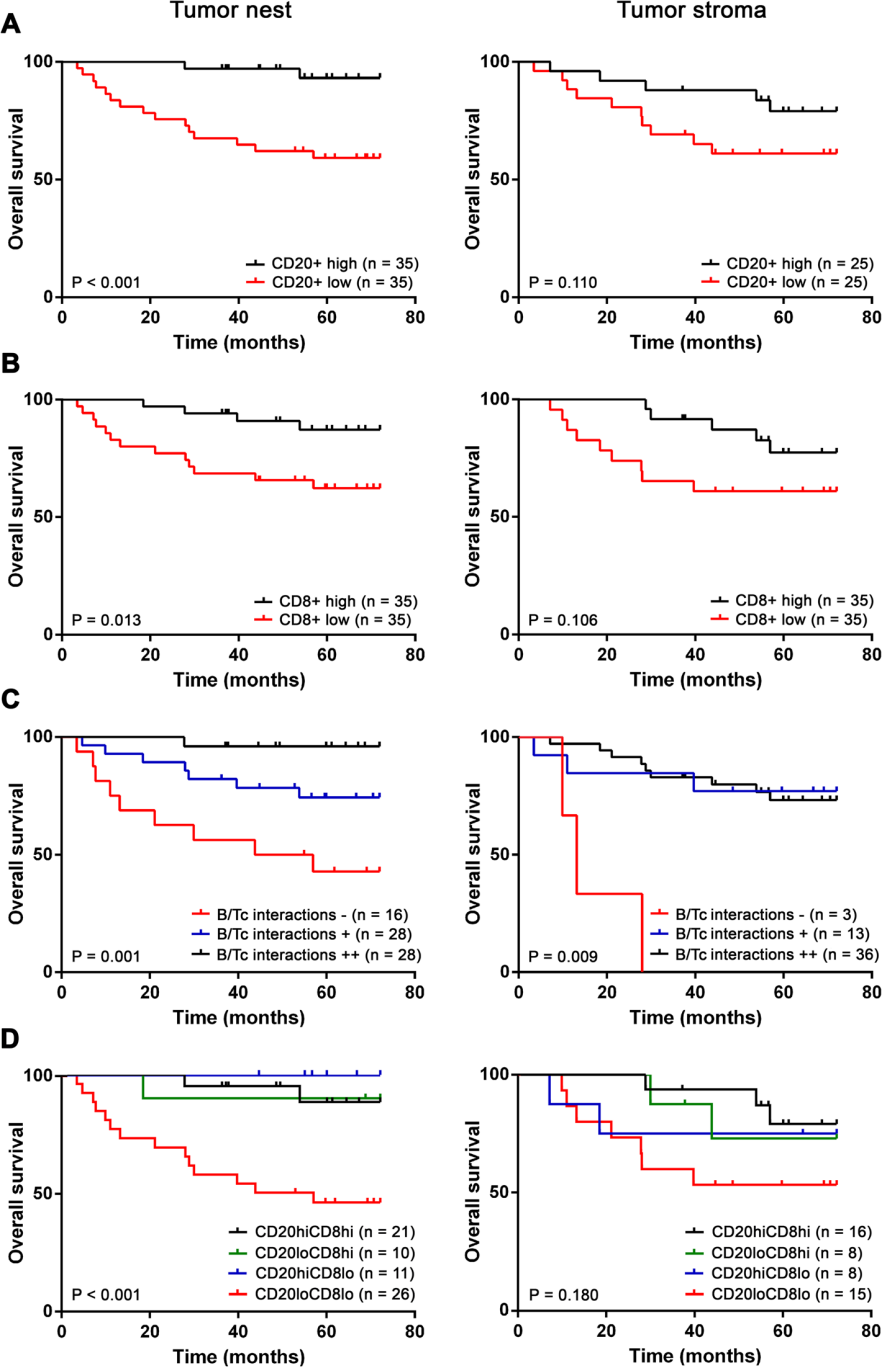
Prognostic value of tumor-infiltrating CD20^+^ B cells (**a**), CD8^+^ T cells (**b**), CD20^+^B cell/CD8^+^ T cell (B/Tc) interactions (**c**) and combination of CD20^+^ B cells and CD8^+^ T cells (**d**) in patients with OPSCC (*n* = 70). Kaplan-Meier curves show overall survival of patients according to densities of the indicated cells in the tumor nests (left) and in the tumor stroma (right). *P* values were determined using the log-rank test

To evaluate the prognostic impact of tumor-infiltrating CD20^+^ B cells, CD8^+^ T cells, DC-LAMP^+^ DCs and B cell/CD8^+^ T cell interactions in both intratumoral and stromal compartments of OPSCC samples, we investigated overall survival (OS) upon stratifying the patient cohort based on the median of positive cells per 1 mm^2^ of the tumor nest and the tumor stroma area. The presence of abundant intratumoral CD20^+^ B cells and CD8^+^ T cells was associated with significantly improved OS (*p* < 0.001 and *p* = 0.013, respectively; Fig. 2a, b). Furthermore, the presence of abundant intratumoral and stromal CD20^+^ B cell/CD8^+^ T cell interactions was also positively correlated with OS. This correlation was highly statistically significant (*p* = 0.001 and *p* = 0.009, respectively; Fig. 2c). Surprisingly, the density of direct CD20^+^ B cell/CD8^+^ T cell interactions stratified the patients better than the concurrent presence of both CD20^+^ B cells and CD8^+^ T cells (Fig. 2d).

Univariate Cox regression confirmed these results, together with well-described risk factors for HNSCC patients, namely, stage IV (*p* = 0.004), extranodal extension (*p* < 0.001), keratinizing histological subtype (*p* = 0.006), advanced tumor size (*p* = 0.042) and HPV negativity (*p* = 0.006). The results are summarized in Table 2. The multivariate Cox proportional hazard model indicated extranodal extension (*p* = 0.004, HR = 5.25, 95% CI = 1.68–16.38), high abundance of CD20^+^ B cells in tumor nests (*p* = 0.044, HR = 0.97, 95% CI = 0.93–0.99) and high abundance of stromal B cell/CD8+ T cell interactions (*p* = 0.019, HR = 0.10, 95% CI = 0.02–0.69) as independent prognostic factors (Additional file 2: Table S2). HPV negativity and high abundance of B cell/CD8^+^ T cell interactions in the tumor nests did not reach statistical significance, but there was a strong trend (*p* = 0.063, HR = 0.29, 95% CI = 0.08–1.06 and *p* = 0.068, HR = 0.11, 95% CI = 0.01–1.17; respectively). The 5-year overall survival (OS) of the patients was 75.7% for the entire Cohort 1 and the median OS was 5.44 years (0.29–14.40).

**Table 2:** Prognostic overall survival parameters in univariate analysis

Variable	Class	Hazard Ratio	95% Confidence Interval	*P* value
Sex	Female	1		
	Male	1.14	0.37–3.50	0.816
Stage	I	1		
	II	2.78	0.78–9.87	0.113
	III	3.51	0.71–17.4	0.124
	IV	7.78	1.92–31.5	**0.004**
LN ratio		2.72	0.44–16.89	0.283
Extranodal extension	No	1		
	Yes	6.55	2.46–17.41	**< 0.001**
Perineural spread	No	1		
	Yes	2.10	0.68–6.47	0.194
Resection margin	R0	1		
	R1	1.56	0.57–4.29	0.389
Concomitant chemotherapy	No	1		
	Yes	1.63	0.62–4.28	0.323
Typing SCC	NK	1		
	NK-M	2.31	0.78–6.90	0.131
	K	5.64	1.64–19.41	**0.006**
Tumor size		1.02	1.00–1.06	**0.042**
HPV status	Negative	1		
	Positive	0.23	0.08–0.66	**0.006**
Smoking history	Non-smoker	1		
	Ex-smoker	0.42	0.13–1.42	0.165
	Smoker	0.63	0.20–1.91	0.412
TLS	No	1		
	Yes	1.15	0.40–3.27	0.789
CD20+ B cell density tumor nest		0.96	0.93–0.99	**0.015**
CD20+ B cell density tumor stroma		1.00	0.99–1.00	0.784
CD8+ T cell density tumor nest		0.99	0.99–1.00	**0.013**
CD8+ T cell density tumor stroma		0.99	0.99–1.00	0.231
DC density tumor nest		0.95	0.88–1.03	0.207
DC density tumor stroma		0.98	0.95–1.02	0.328
B cell/T cell clusters tumor nest	-	1		
	+	0.35	0.13–0.95	**0.040**
	++	0.05	0.01–0.41	**0.005**
B cell/T cell clusters tumor stroma	-	1		
	+	0.07	0.01–0.41	**0.003**
	++	0.08	0.02–0.33	**< 0.001**

Statistically significant *P* values are printed in boldface. Abbreviations: *LN* lymph node, *SCC* squamous cell carcinoma, *NK* non-keratinizing, *K* keratinizing, NK-M non-keratinizing with maturation, *TLS* tertiary lymphoid structures

### In HPV-associated tumors, the presence of CD20^+^ B cell/CD8^+^ T cell interactions positively correlates with the presence and abundance of HPV16 E6/E7-specific CD8^+^ TILs

**Fig. 3:**
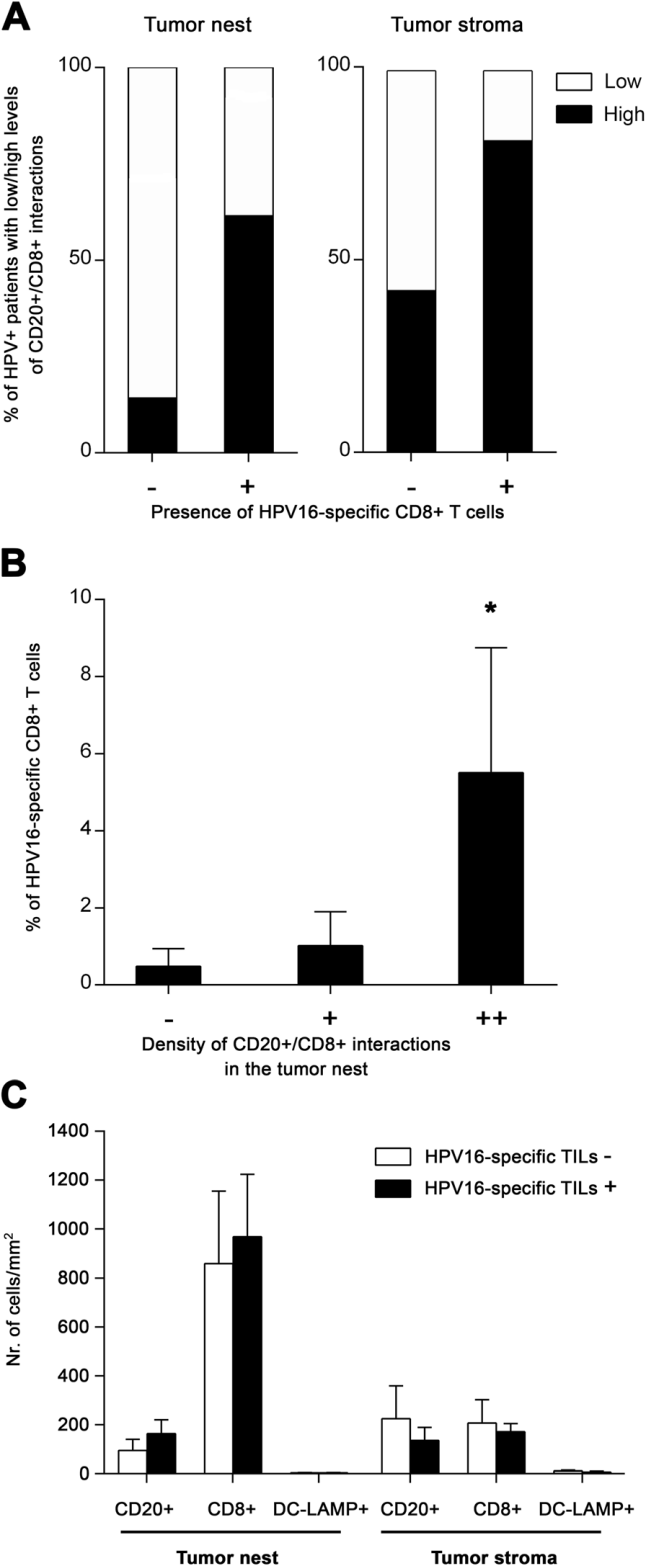
Positive correlation of direct CD20^+^ B cell/CD8^+^ T cell interactions with HPV16 E6/E7-specific CD8^+^ T cells. **a** Columns show the proportions of patients with low (interactions detectable in 0–5 visual fields) and high (interactions detectable in > 5 visual fields) densities of B cell/CD8^+^ T cell interactions with respect to the presence or absence of tumor-infiltrating HPV16 E6/E7-specific CD8^+^ T cells. **b** Columns represent the mean (+ SEM) proportions of tumor-infiltrating HPV16 E6/E7-specific CD8^+^ T cells with respect to the densities of B cell/CD8^+^ T cell interactions within the tumor nests. **c** Columns represent the mean (+ SEM) densities of CD20^+^ B cells, CD8^+^ T cells and DC-LAMP^+^ dendritic cells in tumor nests and tumor stroma of patients without/with detected HPV16-specific T cells. *, *p* < 0.05 (Pearson’s chi-square test and Mann-Whitney U test)

In addition to the differences detected between HPV-positive and HPV-negative tumors, we observed substantial variability in the density of tumor-infiltrating lymphocytes and CD20^+^ B cell/CD8^+^ T cell interactions within the group of patients with HPV-associated tumors, splitting HPV-positive samples into “hot” and “cold” subgroups. Therefore, to assess whether the interactions between CD20^+^ B cells and CD8^+^ T cells might be important for the HPV-specific T cell response in HPV-driven tumors, we correlated the presence and density of B cell/CD8^+^ T cell interactions in the FFPE tumor sections with the proportions of HPV16 E6/E7-specific CD8^+^ T cells detected in TILs expanded from matched native HPV-positive OPSCC samples (Cohort 2). Indeed, 81.8% of patients with detected HPV16 E6/E7-specific CD8^+^ T cells had a high density of B cell/CD8^+^ T cell interactions in the tumor stroma and 61.5% of these patients also had high density of these interactions in the tumor nests. In contrast, it was only 42.8 and 14.3%, respectively, in patients without detected HPV16 E6/E7-specific CD8^+^ T cell responses (Fig. 3a). Moreover, the proportion of HPV16 E6/E7-specific CD8^+^ T cells was significantly positively correlated with the density of B cell/CD8^+^ T cell interactions in the tumor nests (Fig. 3b), indicating that patients with low levels of direct B cell – CD8^+^ T cell interactions also had low levels of HPV16 E6/E7-specific CD8^+^ T cells. On the contrary, the presence of HPV16-specific CD8^+^ T cells was neither correlated to the density of CD8^+^ T cells in general nor to the density of CD20^+^ B cells (Fig. 3c).

### Intratumoral B cells are represented mainly by a memory subtype with an activated, antigen-experienced phenotype

**Fig. 4:**
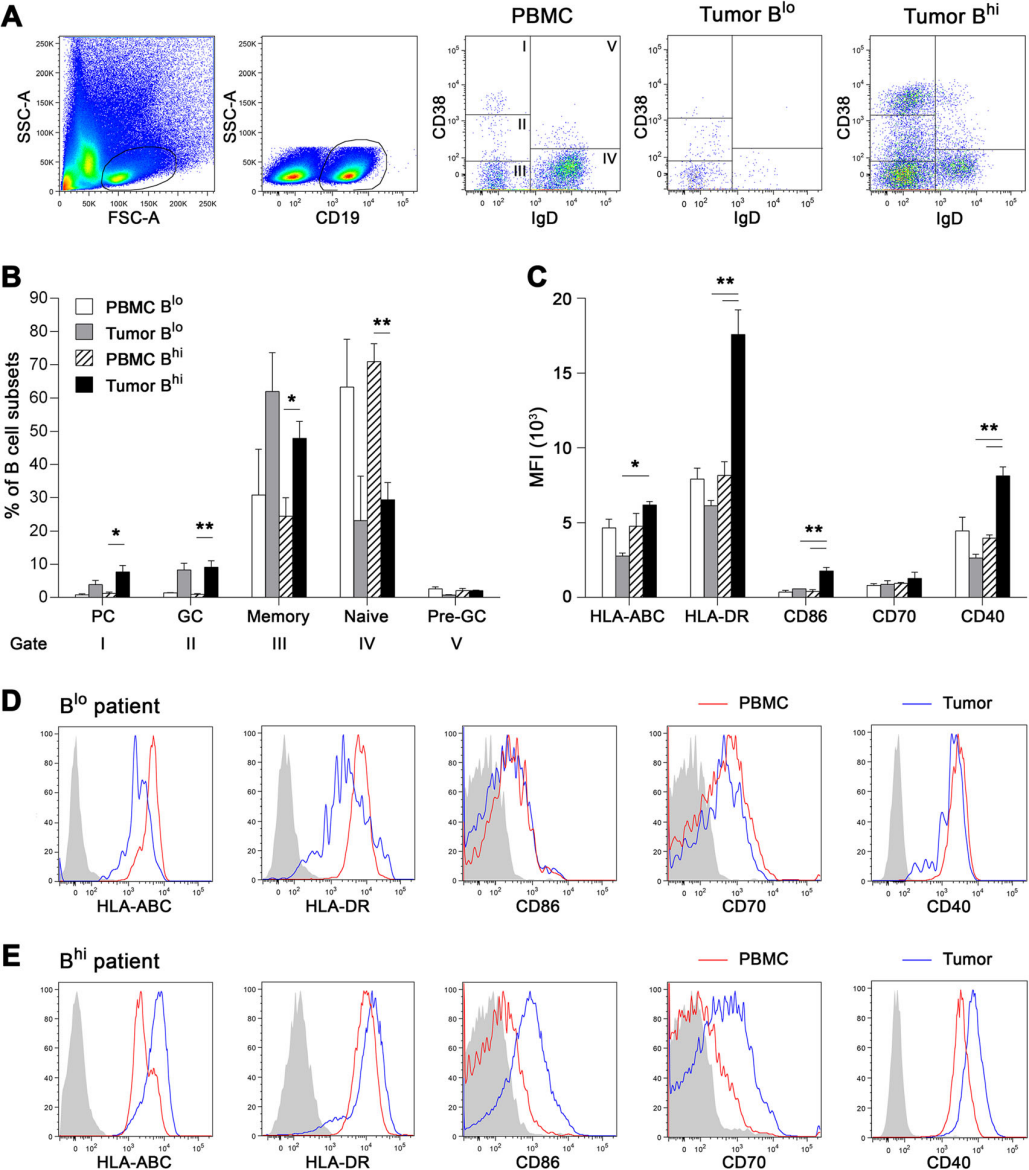
Flow cytometric analysis of tumor-infiltrating B cells and B cells derived from patients’ PBMC divided according to the proportion of TIL-Bs into B^lo^ (% of TIL-Bs < 0.5 of total cells) and B^hi^ samples. Data are expressed as (**a**) representative dot plots and (**b**) mean + SEM of the proportions of B cell subsets within total CD19^+^ B cells. IgD^−^CD38^++^, plasma cells; IgD^−^CD38^+^, germinal center B cells; IgD^−^CD38^−^, memory B cells; IgD^+^CD38^−^, naive B cells; IgD^+^CD38^+^, pre-germinal center B cells. **c** Columns represent mean + SEM of the MFI of B cell surface markers assessed on total CD19^+^ B cells. **d**, **e** Histograms show a representative expression of indicated B cell surface markers in B^lo^ (upper line) and B^hi^ (lower line) patient. Gray-filled areas represent isotype-matched controls, red line represents peripheral blood B cells and blue line represents tumor-infiltrating B cells of the same patient. *, *p* < 0.05; **, *p* < 0.01 (ANOVA followed by Tukey’s post-hoc test)

To characterize the phenotype and function of TIL-Bs in HPV-associated tumors with a “hot” versus “cold” phenotype, we analyzed intratumoral and blood-derived B cell subsets by flow cytometry (Cohort 3). Tumor suspensions were divided according to the proportions of TIL-Bs into “cold” B^lo^ samples (B cell proportions < 0.5% of total cells; mean = 0.11 ± 0.05%) and “hot” B^hi^ samples (mean = 4.22 ± 5.96%). In all samples, CD19^+^ B cells were divided into five subtypes based on the expression level of IgD and CD38, namely, IgD^−^CD38^++^ plasma cells, IgD^−^CD38^+^ germinal center B cells, IgD^−^CD38^−^ memory B cells, IgD^+^CD38^−^ naive B cells and IgD^+^CD38^+^ pre-germinal center B cells (Fig. 4a). Memory B cells represented the major B cell subtype in the tumor tissue (Fig. 4b). There was no difference in the B cell subtype composition between B^lo^ and B^hi^ samples.

Tumor-infiltrating memory B cells were characterized in both B^lo^ and B^hi^ samples by the high expression of CD27, absent expression of IgD and low expression of IgM, indicating a classical memory, predominantly class-switched phenotype. The positivity for the proliferation marker Ki67 in TIL-Bs derived from B^hi^ samples was comparable to that in CD19^+^ B cells derived from healthy tonsils and significantly higher than that in peripheral blood B cells. The proportion of Ki67^+^ TIL-Bs derived from B^lo^ samples was markedly lower in comparison to B^hi^ samples (Additional file 3: Figure S1).

To elucidate whether TIL-Bs might serve as APCs with costimulatory potential, we assessed the expression levels of HLA molecules and costimulatory molecules CD86, CD70 and CD40 on the cell surface. The expression levels of HLA-ABC, HLA-DR, CD86 and CD40 were significantly higher in TIL-Bs derived from B^hi^ OPSCC samples than in TIL-Bs from B^lo^ samples. Additionally, compared to matched peripheral blood B cells, in TIL-Bs derived from B^hi^ samples but not from B^lo^ samples, we observed significantly higher levels of HLA-DR, CD86 and CD40 (Fig. 4c, d, e).

### The presence of B cells in the tumor-derived cell suspension enhances the survival of both CD4^+^ and CD8^+^ TILs

**Fig. 5:**
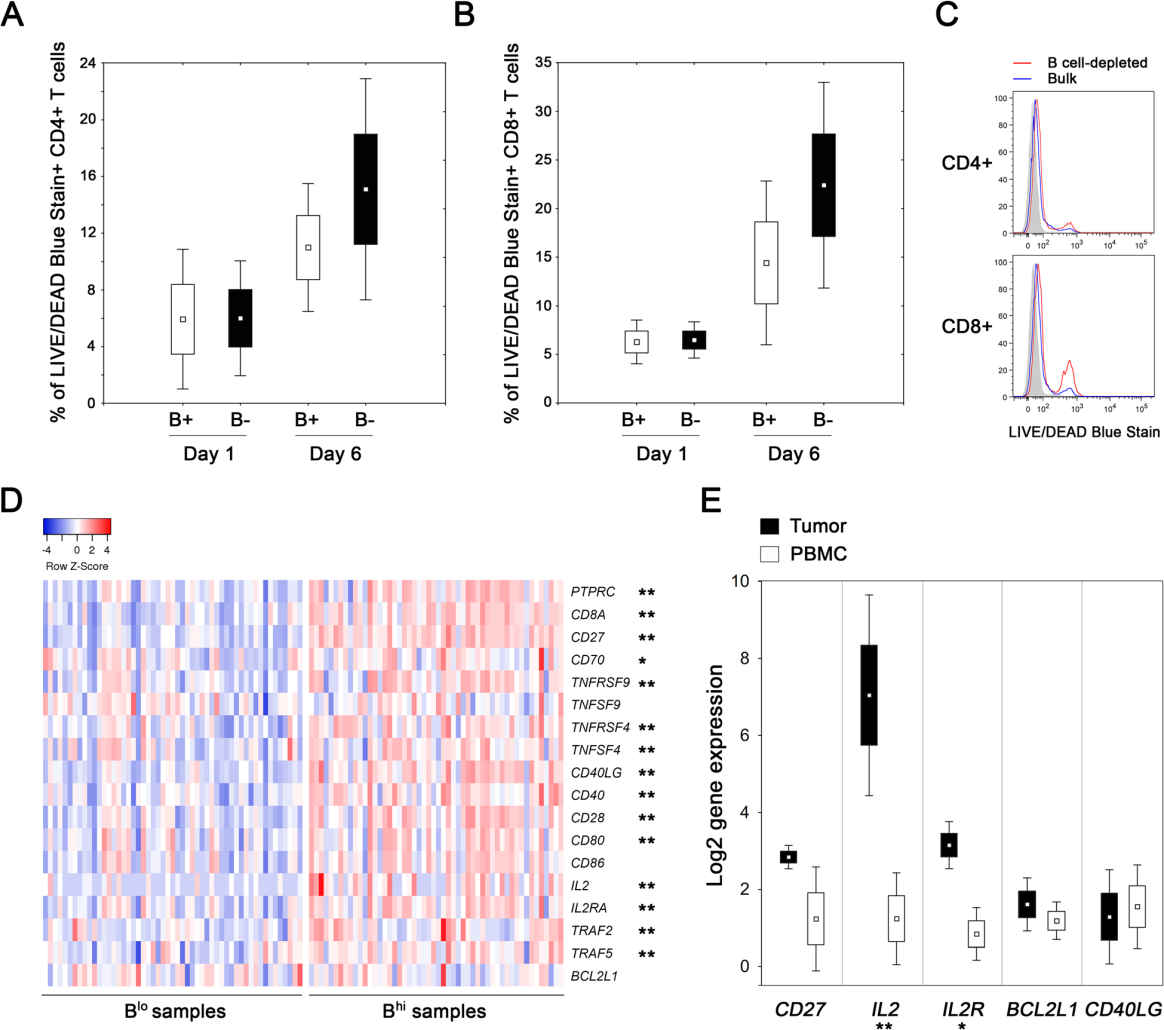
Proportions of dead cells in cultures of bulk and B cell-depleted tumor-derived single cell suspensions. **a**, **b** Box plots show the mean proportion of dead CD4^+^ and CD8^+^ T cells in bulk (B+) and B cell-depleted (B-) tumor-derived cell suspension after 1 and 6 days of cultivation. **c** Histograms show differences in the LIVE/DEAD Blue Stain positivity on day 6 in a representative patient. **d** The heat-map expresses z-scores of relative mRNA expression of indicated genes within B^lo^ (*n* = 53) and B^hi^ (*n* = 52) samples extracted from TCGA databases. **e** Box plots show the mean expression of indicated genes in tumor tissues and matched PBMCs of B^hi^ OPSCC patients (*n* = 4). The boundaries of the box indicate the standard error of the mean and the squares in the box represent the mean. Whiskers indicate the standard deviation. *, *p* < 0.05; **, *p* < 0.01 (t test and Mann-Whitney U test)

To assess the impact of TIL-Bs on survival and functional capacity of T cells, we cultivated B^hi^ tumor-derived cell suspensions and analyzed the viability and cytokine production of CD4^+^ and CD8^+^ T cells after B cell depletion (*n* = 4). In B cell-depleted suspensions, the viability of both CD4^+^ T cells and CD8^+^ T cells did not differ at day 1, but was markedly lower compared to bulk suspensions after 6 days of cultivation without any additional stimuli (15.1 ± 7.8% vs. 11.0 ± 4.5% for CD4^+^ T cells; *p* = 0.068) and 22.4 ± 10.6% vs. 14.4 ± 8.4% for CD8^+^ T cells; *p* = 0.068) (Fig. 5a, b, c). Despite the impaired viability, we did not observe any substantial differences in the proportions of IL-2 and IFN-γ producing CD4^+^ and CD8^+^ T cells with respect to the presence or absence of B cells in the cell cultures.

### Data extracted from TCGA databases confirmed higher expression of costimulatory molecules and IL-2 in B^hi^ HNSCC tumor samples

To estimate the expression levels of a wide spectrum of costimulatory molecules, we analyzed the data extracted from TCGA databases using Statistica® 10.0 software (StatSoft). HNSCC patients with defined p16 status were divided into B^hi^ and B^lo^ subgroups according to the median expression of CD19. With the exception of *BCL2L1*, *TNFSF9* and *CD86*, the B^hi^ samples expressed significantly higher levels of all costimulatory molecules and molecules associated with activation of the TNFR family signaling pathways tested (Fig. 5d).

### CD8^+^ TILs isolated from B^hi^ tumor samples express high levels of IL-2 and IL-2R

In mouse models of viral infections, CD27 was assessed as the key factor in directing the autocrine production of IL-2 that is required for the long-term survival of CD8^+^ T cells in nonlymphoid tissues [35]. Therefore, we analyzed the levels of expression of *IL-2*, *IL-2RA* and *CD27* together with *CD40LG* and the anti-apoptotic regulator *BCL2L1* on CD8^+^ TILs isolated from peripheral blood and tumor tissues of B^hi^ OPSCC patients (*n* = 4; Cohort 3). Indeed, significantly higher levels of *IL-2* and *IL-2R* were expressed in tumor-derived CD8^+^ T cells than in matched peripheral blood CD8^+^ T cells (Fig. 5e).

### IL-10-producing Bregs do not accumulate in B^hi^ OPSCC tumor tissue

Regulatory B cells (Bregs) are characterized by the production of IL-10. To assess the proportion of Bregs in the OPSCC tumor microenvironment, we analyzed the level of IL-10 secreting TIL-Bs after 5 and 24 h of stimulation with CpG ODN 2006 and CD40L in the presence of PMA, ionomycin and brefeldin A using flow cytometry (Cohort 3).

After 5 h of stimulation, the proportion of Bregs, which were found to be predominantly CD5^+^CD24^hi^, was slightly higher in the tumor tissue (0.98 ± 0.78%) compared to matched peripheral blood B cells (0.46 ± 0.12%) and control tonsils (0.41 ± 0.09). Surprisingly, the proportion of IL-10-secreting Bregs after 24 h of in vitro maturation with CpG ODN 2006 and CD40L was significantly lower in the tumor samples than in matched peripheral blood B cells (2.74 ± 0.53% vs. 8.01 ± 1.75%, respectively; *p* = 0.039), but similar to the levels of Bregs in control tonsils (2.16 ± 1.51%). During the longterm stimulation by TLR ligands and CD40L, Breg progenitors mature into IL-10 producing Bregs [37]; therefore, both Bregs and Breg progenitors were detected after 24 h of cultivation in vitro. Interestingly, the proportion of Bregs was negatively correlated to the frequency of CD19^+^ B cells in general (*r* = − 0.69; *p* = 0.085). Due to a limited number of cells, IL-10 production was assessed in B^hi^ samples only.

### B cells are an important source of CXCL9 in the tumor microenvironment

To estimate the impact of TIL-Bs on cytokine production in the tumor microenvironment, we analyzed the spontaneous production of cytokines and chemokines in B^lo^ and B^hi^ tumor-derived cell suspensions and in B^hi^ suspensions depleted of CD19^+^ B cells (Cohort 3). B^hi^ cell suspensions produced markedly higher levels of CXCL9 than B^lo^ cell suspensions (Additional file 4: Figure S2A). In accordance with these results, we observed significantly lower levels of CXCL9 in B cell-depleted samples than in whole cell suspensions (579.6 ± 262.9 vs. 1238.8 ± 290.6 pg/ml, respectively; *p* = 0.025; Additional file 4: Figure S2B), indicating that TIL-Bs are an important source of this chemokine.

## Discussion

We previously described a markedly different immune profile in HPV-associated tumors compared to OPSCCs of other etiologies, characterized by high infiltrates of CD8^+^ T cells [6], with a substantial proportion of HPV16 E6/E7 specific TILs [36]. Indeed, the role of T cell-mediated antitumor immune responses has been extensively studied in the past decade, and consequently, most of the recent immunotherapeutic approaches have been focused on T cells. However, in HNSCC patients, there is still a large proportion of non-responders to recently approved immunotherapy based on PD-1:PD-L1 blockade. Additionally, conventional curative treatment of locally-advanced disease, although effective in patients with HPV-associated tumors, is accompanied by significant morbidity. Therefore, novel immunotherapy targets and consequent effective therapeutic strategies are still crucially needed for this type of carcinoma.

In contrast to T cells, considerably less is known about tumor-infiltrating B cells. Studies concerning TIL-Bs are inconsistent, and both tumor-promoting as well as tumor-inhibitory functions of B cells were reported in various malignancies, whereas the role of B cells in HNSCC has not been satisfactorily evaluated so far. In this study, we assessed the density, distribution and phenotype of TIL-Bs in FFPE and fresh samples from 3 independent cohorts of OPSCC patients.

In accordance with previously published results [26, 38], we observed a significant difference in the B cell-related gene signature between HPV-associated and HPV-negative tumor samples and confirmed these data by showing significantly higher densities of intraepithelial CD20^+^ B cells in FFPE sections of HPV-associated tumors. Moreover, we observed that CD20^+^ TIL-Bs formed with CD8^+^ T cells non-organized small aggregates with clear cell-cell interactions between TIL-Bs and CD8+ TILs, and both the densities of intraepithelial CD20^+^ B cells and B/CD8^+^ T cell interactions were shown to have prognostic significance for the overall survival of the patients, regardless of the HPV status. In HPV-positive tumors, the formation of B/Tc interactions was also strongly associated with the presence and abundance of HPV16 E6/E7-specific CD8^+^ T cells. Additionally, we observed a significantly higher expression of activation molecules, namely, HLA-ABC, HLA-DR, CD86 and CD40, in TIL-Bs derived from tumor samples with high levels of B cells in comparison to TIL-Bs derived from B^lo^ (< 5% of total cells) samples. Importantly, depletion of B cells led to markedly lower viability of CD4^+^ and CD8^+^ T cells in tumor-derived cell cultures. These data indicate not only quantitative but also qualitative differences in B cell-mediated immune responses between OPSCC patients with high vs. low densities of TIL-Bs.

A positive association between the high density of B cells and prolonged patient overall survival has been previously reported in ovarian cancer [39], hepatocellular carcinoma [24, 40], NSCLC [20] and breast cancer [22, 41]. Whereas in NSCLC, the major importance was assigned to TLS formation and the presence of follicular B cells [20], Nielsen [39] and Garnelo [40] emphasized the cell-to-cell contact of B and T cells within the tumor microenvironment and a positive correlation between B cell and T cell densities. In accordance with the latter studies, we observed the formation of aggregates of B cells and CD8^+^ T cells with clear cell-to-cell contacts in some OPSCC patients with high densities of CD20^+^ B cells. Together with intraepithelial CD8^+^ T cells, a high density of CD20^+^ B cells within the tumor nest and high densities of B/CD8^+^ T cell interactions in both the tumor nest and stroma were confirmed as positive prognostic markers. The Cox proportional hazard model determined the intraepithelial density of CD20^+^ B cells and stromal density of B/CD8^+^ T cell interactions as independent prognostic markers stronger than HPV and CD8^+^ T cell density alone. However, as the proportion of HPV-negative samples was markedly lower compared to HPV-positive samples (12.5% vs. 87.5%, respectively) in our cohort of patients, the impact of HPV status might be underestimated. Most importantly, in an independent cohort of patients with HPV-related tumors, the density of B/CD8^+^ T cell interactions was significantly associated with the proportions of HPV16 E6/E7-specific CD8^+^ T cells, indicating the importance of in situ B cell-CD8^+^ T cell interactions in the antigen-specific antitumor immune response. In contrast, DC-LAMP^+^ DCs occurred at markedly lower densities than TIL-Bs and were neither correlated with patient prognosis nor the abundance of HPV16 E6/E7-specific CD8^+^ T cells.

In addition to differences in CD20^+^ B cell and CD8^+^ T cell densities in HPV-positive and HPV-negative OPSCC samples, we observed substantial variability in the levels of tumor-infiltrating immune cells in patients with HPV-associated tumors, with a clear subgroup of immunologically “cold” HPV-positive tumors. Therefore, we analyzed the phenotype of TIL-Bs in fresh HPV-positive tumor samples with high vs. low infiltrates of B cells. In contrast to ovarian and hepatocellular carcinomas [24, 39], but in accordance with the study published by Lechner et al. [26], OPSCC-derived TIL-Bs showed a classical memory phenotype with high expression of CD27 and low/no expression of CD38, IgD and IgM. Importantly, we observed substantial differences in TIL-Bs derived from highly infiltrated samples and samples with markedly low (< 0.5% of total cells) B cell densities. In B^hi^ samples, TIL-Bs showed an activated phenotype with high levels of HLA-ABC, HLA-DR, CD86 and CD40, whereas the expression of activation markers in TIL-Bs from B^lo^ samples was significantly lower. Additionally, the proportion of proliferating Ki-67^+^ TIL-Bs was markedly higher in B^hi^ compared to B^lo^ samples, further indicating a low level of B cell activation in B^lo^ tumors. These data show that the substantial difference between B^hi^ patients with anticipated good outcome and B^lo^ patients with poor outcome may not entirely be due to the B cell quantity but may reflect the distinction in phenotype and consequent functional capacity of TIL-Bs.

In accordance with the study focused on tongue squamous cell carcinoma [42], we have observed higher frequency of CD19^+^IL-10^+^ Bregs in the tumor tissue compared to peripheral blood and control tonsils; however, the difference did not reach statistical significance, probably due to a high variability within the tumor group. Interestingly, the abundance of Bregs markedly negatively correlated with the frequency of bulk CD19^+^ B cells, suggesting that a high level of Bregs might be associated with tumor samples with low level of B cell infiltration in general, which mostly show immunologically “cold” phenotype with low densities of CD8^+^ T cells. This could also explain the discrepancy between our study and the study by Lechner et al. [26], who observed very high proportions of Bregs in mainly HPV-negative, i.e. most probably immunologicaly “cold” HNSCC samples. These data nevertheless need further examination employing a larger cohort of patients, including tumor samples with both high and very low frequencies of B cells (< 0.5% of total cells).

The colocalization of CD20^+^ B cells with CD8^+^ T cells, the association of these cell-cell interactions with the presence and frequency of HPV16 E6/E7 CD8^+^ T cells and the highly activated phenotype of TIL-Bs derived from B^hi^ samples leads to two possible mechanisms for how CD20^+^ TILs may promote T cell-mediated immune responses. First, B cells are capable of producing chemokines, such as CXCL9, and cytokines, such as lymphotoxin, which recruit T cells to the tumor tissue and promote the formation of local lymphoid structures [20, 39]. Indeed, our data suggest that TIL-Bs may be an important source of CXCL9, a potent T cell chemoattractant [43]. In contrast, we did not detect lymphotoxin production in OPSCC tumor tissue-derived cell suspensions, which is in accordance with low levels of classical TLS observed in OPSCC FFPE sections.

Second, TIL-Bs may serve as local APCs, permitting the long-term persistence of antigen-specific CD8^+^ T cells in the tumor microenvironment [29]. Indeed, we observed a substantial decrease in both CD4^+^ T cell and CD8^+^ B cell viability after depletion of TIL-Bs from tumor-derived cell suspensions. Additionally, we found exceptionally high levels of CD40 on TIL-Bs originating from B^hi^ tumor samples. CD40L principally expressed on activated T cells interacts with CD40, leading to a “licensed” state of APCs [44]. Licensed APCs upregulate the expression of costimulatory molecules, which further interact with mediators of T cell activation from the TNF receptor family, including CD27, 4-1BB and OX40 [45]. Importantly, CD40 stimulation promotes cross-priming of exogenous antigens in APCs, resulting in efficient CD8^+^ T cell stimulation [46, 47]. In models of viral infections, the accumulation and survival of virus-specific CD8^+^ T cells at the tissue site relied strongly on CD27/CD70 and to a lesser extent on 4-1BB and OX40 signaling [33, 35]. Interestingly, Peperzak et al. [35] demonstrated that the survival of effector CD8^+^ T cells in nonlymphoid tissue of influenza-infected mice is directed mainly by CD27/CD70-mediated autocrine production of IL-2.

In accordance with these studies, using data from TCGA databases we observed significantly higher levels of *CD40*, *CD40LG*, *CD27*, *CD70*, *TNFRSF4* (OX40), *TNFSF4* (OX40L), *TRAF2*, *TRAF5*, *IL-2* and *IL-2RA* expression in B^hi^ samples compared to B^lo^ HNSCC tumors. Importantly, we showed that in comparison to matched peripheral blood CD8^+^ T cells, CD8^+^ TILs express significantly higher levels of *IL-2* and *IL-2RA*. Therefore, we suggest that in B^hi^ tumors, TIL-Bs might recruit CD8^+^ T cells via CXCL9 and crucially contribute to the survival of the CD8^+^ T cells in the tumor microenvironment due to the in situ secondary costimulation employing CD40L/CD40 and TNFR/TNF superfamily signaling pathways.

## Conclusions

This study provides an extensive analysis of B cells in the OPSCC microenvironment, highlighting intraepithelial TIL-Bs as a valid prognostic marker, which surpasses the confirmed biomarkers such as HPV positivity and CD8^+^ TIL density in stratification of OPSCC patients. Thus, the density of B cells and/or the density of direct B cell/CD8^+^ T cell interactions may help to preselect patients with excellent prognosis who would profit from less invasive treatment and consequently decreased toxicity of the therapy. Additionally, our study suggests that in OPSCC, TIL-Bs might provide costimulatory signals important for CD8^+^ T cell maintenance in the tumor tissue. Consequently, including B cells as an additional target into novel immunotherapeutic protocols may help to establish sustained antitumor T cell responses in situ and thus improve current approaches mainly focused on T cell (re)stimulation alone. However, as all of the patients in our cohorts received surgery as the main therapeutic option, the application of reported results to patients receiving primary curative chemoradiotherapy needs to be further analyzed.

## Additional files

Additional file 1: **Table S1.** List of monoclonal antibodies used for flow cytometry. (DOCX 16 kb)

Additional file 2: **Table S2.** Prognostic overall survival parameters in multivariate analysis. (DOCX 13 kb)

Additional file 3: **Figure S1.** Activation markers in TIL-Bs. (A) Representative figures show expression of CD27 and IgM in CD38^−^IgD^−^ memory B cells (red line) and CD38^−^IgD^+^ naive B cells (blue line). (B) Columns show the mean proportion of Ki67^+^CD19^+^ B cells in peripheral blood and tumor tissue of B^hi^ (proportion of TIL-B cells > 0.5% of total cells) and B^lo^ OPSCC patients and control healthy tonsils. Whiskers represent the standard error of mean (SEM). *p < 0.05 (Kruskal-Wallis ANOVA). (PDF 1084 kb)

Additional file 4: **Figure S2.** Cytokine and chemokine profiles of tumor-derived single cell suspensions. (A) White columns represent the mean spontaneous cytokine production in B^lo^ samples (*n* = 3); black columns represent cytokine production in B^hi^ samples (*n* = 7). (B) Black columns represent the mean spontaneous cytokine/chemokine production by whole B^hi^ tumor-derived single cell suspensions; white columns represent B cell depleted cell suspensions. All error bars represent SEM. * *p* < 0.05 (paired t-test). (PDF 409 kb)

## Data Availability

The datasets used and/or analyzed during the current study are available from the corresponding author on reasonable request.
