# Supplementary material for: Tumor-infiltrating B cells affect the progression of oropharyngeal squamous cell carcinoma via cell-to-cell interactions with CD8+ T cells
Source: J Immunother Cancer. 2019 Oct 17;7:261. doi: 10.1186/s40425-019-0726-6 (PMC6796441; doi:10.1186/s40425-019-0726-6)
Supplement: Supplementary file 1 — Table S1. List of monoclonal antibodies used for flow cytometry. (DOCX 16 kb) [file 40425_2019_726_MOESM1_ESM.docx]

**Supplementary data**

**Table S1: List of monoclonal antibodies used for flow cytometry**

| Antigen | Fluorochrome | Clone | Producer |
| --- | --- | --- | --- |
| CD8a | PE-DyLight 594 | MEM31 | Exbio |
| CD19 | FITC | LT19 | Exbio |
| CD21 | PerCP-Cy 5.5 | Bu32 | Biolegend |
| CD27 | APC | M-T271 | Biolegend |
| CD38 | Alexa Fluor 700 | HB-7 | Biolegend |
| CD40 | Brilliant Violet 421 | 5C3 | Biolegend |
| CD70 | PE | Ki-24 | BD Biosciences |
| CD86 | PE | HA5.2B7 | Immunotech |
| HLA-ABC | Alexa Fluor 700 | W6/32 | Biolegend |
| HLA-DR | Brilliant Violet 421 | L243 | Biolegend |
| IgD | Brilliant Violet 421 | IA6-2 | Biolegend |
| IgM | PerCP-Cy 5.5 | MHM-88 | Biolegend |
| IL-10 | PE | JES3-9D7 | Biolegend |
| Ki-67 | Pe-Cy 7 | Ki-67 | Biolegend |
