# Supplementary material for: Tumor-infiltrating B cells affect the progression of oropharyngeal squamous cell carcinoma via cell-to-cell interactions with CD8+ T cells
Source: J Immunother Cancer. 2019 Oct 17;7:261. doi: 10.1186/s40425-019-0726-6 (PMC6796441; doi:10.1186/s40425-019-0726-6)
Supplement: Supplementary file 2 — Table S2. Prognostic overall survival parameters in multivariate analysis. (DOCX 13 kb) [file 40425_2019_726_MOESM2_ESM.docx]

| Table S2: Prognostic overall survival parameters in multivariate analysis | | | | |
| --- | --- | --- | --- | --- |
| Variable | **Class** | **Hazard Ratio** | **95% Confidence Interval** | **P value** |
| Extranodal extension | No  Yes | 1  5.25 | 1.68-16.38 | **0.004** |
| Tumor size |  | 0.99 | 0.96-1.02 | 0.592 |
| HPV status | Negative  Positive | 1  0.29 | 0.08-1.06 | 0.063 |
| CD20+ B cell density  tumor nest |  | 0.97 | 0.94-1.00 | **0.044** |
| CD8+ T cell density  tumor nest |  | 1.00 | 1.00-1.00 | 0.581 |
| B cell/T cell clusters  tumor nest | -  +  ++ | 1  0.59  0.11 | 0.13-2.65  0.01-1.17 | 0.491  0.068 |
| B cell/T cell clusters  tumor stroma | -  +  ++ | 1  0.10  0.24 | 0.02-0.69  0.05-1.19 | **0.019**  0.081 |

Abbreviations: SCC, squamous cell carcinoma; NK, non-keratinizing; K, keratinizing; NK-M, non-keratinizing with maturation.
