# Supplementary figures and images for: Tumor-infiltrating B cells affect the progression of oropharyngeal squamous cell carcinoma via cell-to-cell interactions with CD8+ T cells
Source: J Immunother Cancer. 2019 Oct 17;7:261. doi: 10.1186/s40425-019-0726-6 (PMC6796441; doi:10.1186/s40425-019-0726-6)

**A**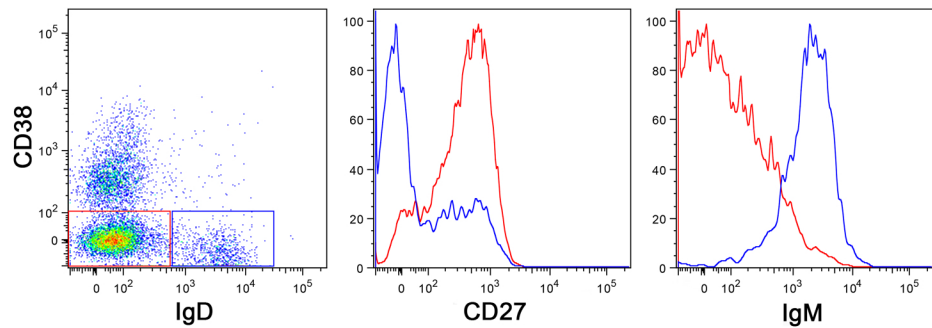**B**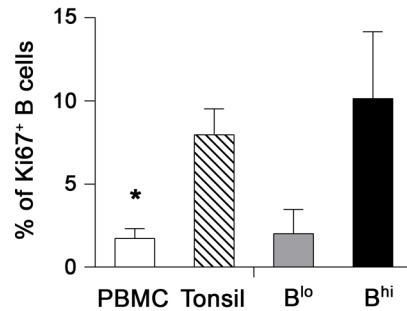

Supplement: Supplementary file 3 — Figure S1. Activation markers in TIL-Bs. (A) Representative figures show expression of CD27 and IgM in CD38−IgD− memory B cells (red line) and CD38−IgD+ naive B cells (blue line). (B) Columns show the mean proportion of Ki67+CD19+ B cells in peripheral blood and tumor tissue of Bhi (proportion of TIL-B cells > 0.5% of total cells) and Blo OPSCC patients and control healthy tonsils. Whiskers represent the standard error of mean (SEM). *p < 0.05 (Kruskal-Wallis ANOVA). (PDF 1084 kb) [file 40425_2019_726_MOESM3_ESM.pdf]

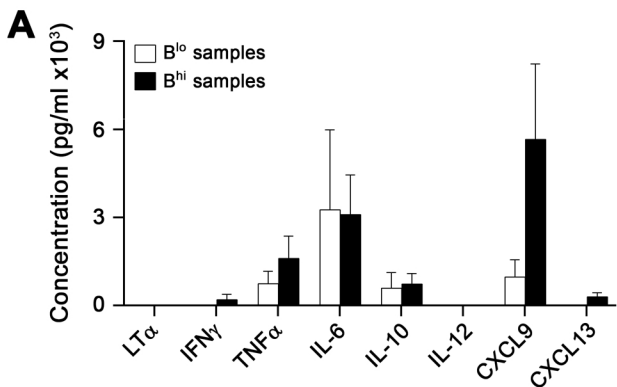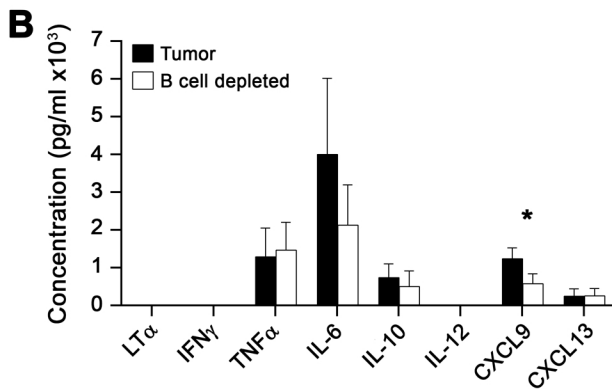

Supplement: Supplementary file 4 — Figure S2. Cytokine and chemokine profiles of tumor-derived single cell suspensions. (A) White columns represent the mean spontaneous cytokine production in Blo samples (n = 3); black columns represent cytokine production in Bhi samples (n = 7). (B) Black columns represent the mean spontaneous cytokine/chemokine production by whole Bhi tumor-derived single cell suspensions; white columns represent B cell depleted cell suspensions. All error bars represent SEM. * p < 0.05 (paired t-test). (PDF 409 kb) [file 40425_2019_726_MOESM4_ESM.pdf]
